# Gender equality among medical and dental academic researchers in West Africa: a theoretical analysis of a compendium of research

**DOI:** 10.3389/froh.2025.1373404

**Published:** 2025-02-21

**Authors:** Moréniké Oluwátóyìn Foláyan, Ana Gascón-Catalán, Guillermo Z. Martínez-Pérez

**Affiliations:** ^1^ Department of Physiatrics and Nursing, Faculty of Health Sciences, University of Zaragoza, Zaragoza, Spain; ^2^ Oral Health Initiative, Nigerian Institute of Medical Research, Lagos, Nigeria; ^3^ Department of Child Dental Health, Obafemi Awolowo University, Ile-Ife, Nigeria; ^4^ Africa Oral Health Network, University of Alexandria, Alexandria, Egypt

**Keywords:** inequality, gender, theoretical frameworks, leadership, research productivity, West Africa, Nigeria

## Abstract

**Background:**

This study conducted theoretical analyses of the findings of a study on gender disparities in research productivity and leadership in medical and dental research institutions in Nigeria. The aim was to highlight the connectedness of the study findings, and to develop a conceptual framework that can inform future studies on gender equity in academia across West Africa.

**Methods:**

A content analysis of the research outputs was conducted using four theoretical frameworks to assess the alignment of the study findings with gender equality goals. The research examined policy effectiveness and institutional practices using the 2008 USAID Gender Equality Framework; analyzed how personal traits, societal expectations, and organizational practices intersect using Fagenson's Theory of Gender and Career Development; uncovered gendered power dynamics and inform strategies for institutional reform using the Feminist Institutionalism Analytical Lens; and shed light on disparities in mentorship, collaboration, and academic publishing using the Academic Literacies Theory.

**Results:**

The key domains influencing gender inequality and requiring targeted actions are: first, the patriarchal societal, cultural, and religious values that shape gender roles, restricting women's career advancement. Second, institutional policies and organizational culture that limits female leadership and research productivity. Third, individual and generational perspectives influence advocacy, with younger individuals recognizing inequality more readily. Research productivity and leadership disparities can be addressed through mentorship and training programs for early-career researchers that foster the development of gender-sensitive advocates. The developed conceptual framework outlines three core research and action domains—societal, institutional, and individual factors—and their sub-factors, alongside targeted interventions influencing gender inequality, proposed targeted recommendations and expected outcomes.

**Conclusions:**

The findings emphasize the multifaceted nature of gender disparities. Strategic actions are needed to address the issues that foster gender equality in medical and dental research institutions, and to support female researchers in West Africa. These actions should focus on the younger generations to drive needed changes.

## Introduction

In Nigeria, there were 86,885 non-academic male staff and 51,671 academic male staff in the 2018/2019 academic year (1). In contrast, the female workforce consisted of 46,869 non-academic and 16,009 academic staff members in the same academic year (1). This gender disparity also extends to medical and dental research institutions. Within this broader gender gap, it is important to recognize that women in health research face disproportionate challenges including a lower sense of belonging compared with their male colleagues, sexual harassment, and lower pay (2–4). To tackle this issue, it is essential to gain an understanding of the factors contributing to gender inequality in research productivity and leadership roles within medical and dental research institutions.

We conducted a project that identified the obstacles limiting the progression of female researchers in Nigeria's academic landscape; explored how researchers perceive gender disparities within the realm of medical and dental research institutions in Nigeria; examined the decision-making processes employed to address and navigate gender inequities within medical and dental research institutions in Nigeria; explored perspectives on the establishment of a conducive environment for female medical and dental researchers; and identified strategies for enhancing the underrepresented presence of women in the field of scientific research (4).

The project was conducted in four phases. First, a series of interviews were conducted with 30 researchers, comprising 21 female researchers and 9 male researchers, who hailed from Ghana, Senegal, Burkina Faso, Niger, and Mali. The age spectrum among the participants spanned from 30 to 56 years, and their research experience encompassed a range of 5 to over 30 years. Four key themes emerged as barriers to women's career development. First, family- and environment-related barriers were highlighted, with gender norms assigning domestic tasks and responsibilities to women, thereby limiting the time they could devote to research. Second, an institutional culture and policies insensitive to gender dynamics were identified, which exacerbated gender disparities and hindered women from achieving leadership positions. Third, there was a call for emancipation programs for women in research to build resilience and enhance critical decision-making, as existing strategies often focused narrowly on addressing spousal relationships rather than broader structural challenges. Finally, individual perceptions of professional and personal success were noted, with many women viewing themselves as equally competent as their male peers, yet still facing persistent gender discrimination (6).

Second, a qualitative study was implemented between March and July 2022 in Nigeria to delve into the perspectives of researchers regarding gender inequalities and disparities in the career progression and trajectory of medical and dental researchers in Nigeria. Data was generated from 54 medical and dental professionals who took part in the in-depth interviews. The sample comprises 48% females and 52% males, 65% of respondents who were younger than 50 years, and 22% had reached the professorial cadre. Three core themes emerged: institutionalized male dominance in research institutions, shifting narratives on gender equality in academia, and women leading the push for change. Female medical and dental researchers are challenging androcentric norms in knowledge production, questioning patriarchal values that contribute to low female enrolment in training, limited research outputs, and underrepresentation in senior leadership roles (7).

The study also identified differences in perspectives based on generational and gender divisions. Among the younger generation, both males and females shared a common view on gender inequality in leadership positions and research opportunities, particularly disadvantaging female academics. They emphasized the necessity for change from a human rights perspective. Conversely, the older generation exhibited divergent opinions. Males acknowledged the presence of gender inequality in leadership and research opportunities, but proposed changes based on commodifying women. Older females adhered to traditional views regarding gender inequality. Recommendations for change included removing barriers to education for girls, which restrict their access to capacity-building opportunities (4).

Third, in June 2022, a bibliometric analysis was carried out to evaluate disparities in productivity, influence, collaboration trends, and authorship roles among researchers in the field of dentistry and oral sciences in Nigeria. Specifically, this involved a search and analysis of articles authored by Nigerian dentists, published between 2012 and 2021, and accessible within the Web of Science database related to dentistry and oral sciences researchers. Data was gathered on gender-based distinctions in research output, academic impact, collaborative patterns, and authorship roles, including first, last and corresponding authorship. We identified 413 unique authors who published a total of 1,222 articles on dentistry and oral sciences in Nigeria between 2012 and 2021. Females had a slightly higher average number of citations per author compared to males, and a higher percentage of papers published by males listed international collaborators and domestic collaborators, but these differences were not statistically significant. Significantly, more females than males were listed as first authors, while a significantly greater percentage of males than females were listed as last authors. Additionally, a slightly higher percentage of females than males were listed as corresponding authors (8).

Fourth, between April and May 2023, a descriptive qualitative study was undertaken to gather data through in-depth interviews involving eight senior female researchers who had previously held leadership positions within medical and dental research institutions in Nigeria. The selection of interviewees was based on a pool of 20 eligible female faculty members at Nigerian universities, who occupy managerial roles, and actively contribute to the promotion, design, execution, and dissemination of biomedical, clinical, and socio-epidemiological research within Nigeria. The explorative study shed further light on the patterns of male dominance within research and academic institutions, the gender disparities in women's entry into such institutions, the necessary environment, and resources to promote women's advancement into managerial positions, and the factors contributing to the observed gender differences in research productivity. While participants acknowledged the increasing presence of females in the medical and dental research academia, there was no consensus that a higher number of first female authorships corresponded to a proportional increase in junior authorships. Patriarchal social practices were identified as potential contributors to the lower participation of females in collaborative research within the dental academia (4).

The findings from this project suggest that gender disparities in research productivity and leadership roles within medical and dental research institutions in West Africa, particularly in Nigeria, are rooted in patriarchal institutional practices, limited access to professional resources, and cultural norms that restrict women's agency. This study aimed to analyze these findings through four theoretical lenses that highlight the impact of patriarchal institutions on advancing gender equality in higher education institutions. The theoretical analysis of the project findings will guide the development of a conceptual framework aimed at driving systemic reforms and targeted empowerment initiatives.

The four theoretical perspectives—the USAID Gender Equality Framework (9), Fagenson's Theory of Gender and Career Development, the Feminist Institutionalism Analytical Lens, and the Academic Literacies Theory—offer complementary yet conflicting approaches to addressing gender disparities in academia. The USAID Gender Equality Framework (9) focuses on structural reforms, advocating for policies and institutional support to ensure equitable access, but it underemphasizes individual agency (10). Fagenson's theory, in contrast, highlights individual career development, mentoring, and networking, prioritizing personal agency within existing systems, potentially clashing with systemic reform approaches (11). Feminist Institutionalism critiques patriarchal systems, calling for structural change to dismantle entrenched inequalities, creating tension with theories like Fagenson's that emphasize adaptation over transformation (12–14). The Academic Literacies Theory highlights implicit norms and power dynamics in academia, aligning with systemic critiques but diverging from frameworks prioritizing resource redistribution by focusing on the hidden curriculum over overt structural change (15, 16).

## Methods

### Ethics approval

Ethical approval for the conduct of first phase was obtained by the Senegalese National Health Research Ethics Committee was obtained for this study (0000050/MSAS/DPRS/CNERS). Ethics approval for the conduct of the study was obtained from the Institute of Public Health, Obafemi Awolowo University, Ile-Ife, Nigeria (IPH/OAU/12/1617).

### Theoretic analyses

A content analysis of the research outputs was conducted to assess their alignment with gender equality goals. Using the USAID Gender Equality Framework (9), the research examined policy effectiveness and institutional practices. Fagenson's Theory of Gender and Career Development guided the analysis of how personal traits, societal expectations, and organizational practices intersect. The Feminist Institutionalism Analytical Lens uncovered gendered power dynamics and inform strategies for institutional reform, while the Academic Literacies Theory shed light on disparities in mentorship, collaboration, and academic publishing. Findings from bibliometric analysis and interviews were triangulated to ensure validity.

## Results and discussion

The study results offer insights into a regional context that elucidates the observed gender disparity in the career trajectories of women in research. This adds valuable context to the global discourse on promoting gender equity in global research career development. Furthermore, the findings from the Nigerian segment of the study support a feminist institutionalist perspective that contends societal inequality is perpetuated within political and social institutions, such as higher education institutions (17). Understanding how context-specific institutional rules, processes, and norms contribute to gender inequality enactment can inform institutional gender equality programs and strategies. This study is the first to explore the how and why of gender inequality enactment in medical and dental schools in Nigeria, thus providing a framework to support potential gender reforms within these institutions.

### Barriers to the career advancement of women researchers in some West African countries

The current study highlights that the gendered roles of women in overseeing domestic and caregiving duties remain significant barriers to their participation and advancement in research. This aligns with Milewski et al. (18), who highlight the challenges women face balancing work and family life, often resulting in precariousness. Our study shows that family responsibilities frequently limit women researchers' time for career development, echoing Sayer's (19) observation that women spend more time on unpaid domestic work than men. In African households, subordinate roles (20) often require women to seek spousal approval for career pursuits, deterring many from entering research. This dynamic may contribute to persistent gender disparities in West African biomedical research careers (21), necessitating further investigation.

Organizational factors also hinder women's career progression. Research institutions in West Africa often reflect societal gender inequalities, with organizational cultures favoring male professors and students (22, 23). Leadership structures and power dynamics are shaped by male expectations, forcing women to navigate both domestic gender norms and institutional biases. These challenges frequently lead women to prioritize societal expectations over career ambitions. Prior studies had identified that male-dominated networks, bullying, and harassment are common challenges women face in male-dominated in male-dominated institutions (24). However, some academic institutions implement programs to promote gender equity, primarily targeting individual and interpersonal interactions (25).

Institutional changes need to be complimented with individual empowerment. Empowerment involves a socio-political process where women become aware of and transform relations of domination. For example, in our study, some women researchers in Togo prefer their husbands take second wives in polygamous unions to delegate domestic tasks and create career time (26). Divorce is less favored, likely due to societal stigma that limits leadership opportunities for unmarried women.^1^ The USAID Gender Equality Framework (9) underscores the importance of addressing individual, structural, and societal factors contributing to these disparities. In addition, the findings align with Fagenson's theory, which highlights the interplay of family dynamics, organizational factors, and career development strategies. Traditional gender roles continue to influence the work-family dynamic, and the disproportionate share of unpaid domestic work limits their time for research and career advancement (18, 19, 27).

Spousal support reduces the likelihood of women leaving the workforce due to domestic pressures (28, 29). Cultural differences in spousal support are notable. In Saudi Arabia, women emphasized the importance of spousal assistance with household tasks over financial support (30), while in India, shared domestic responsibilities and emotional encouragement were key to career advancement (31, 32). Our study on Nigerian medical and dental academia revealed that male involvement in decision-making, and the lack of shared domestic responsibilities constrain women's career advancement. Unlike previous discussions, this study highlights the restricted autonomy of educated, socially advantaged women beyond household needs, calling for an intersectional feminist approach to address these intertwined dimensions of identity and power.

The current study findings highlight the need for further exploration of women's empowerment and emancipation within the context of societal constraints. This imbalance is worsened by limited mentoring, and perceived poor organizational support (33). This imbalance affects their engagement in research activities and hinders career progression highlighting the need for spousal support for career advancement (34). Using the academic literacies theory lens, this struggle aligns with the view of academic writing as a complex skill requiring training (35), emphasizing the need for innovative solutions to support skill acquisition within their contexts. Local traditions must be considered to address cultural barriers to women's career progression. Empowerment should be framed as a multidimensional socio-political process extending beyond academic literacy to navigating complex institutional dynamics.

Strategies to enhance women's involvement in research and academia through policies that increase access to essential resources such as time, support networks, and career development opportunities are needed. Addressing the burden of unpaid domestic work, promoting equitable resource access, and empowering women to manage dual roles and decision-making within the family are critical. Achieving gender equality requires both short-term empowerment initiatives and long-term systemic reforms, tailored to culturally appropriate strategies, to create an inclusive and equitable academic environment.

### Researchers’ perceptions of gender equality enacted in the medical and dental research field in Nigeria

The study also observed that research institutions in West Africa often replicate broader societal gender inequalities, aligning with Fagenson's theory and feminist institutionalism. Male-dominated networks within these institutions create additional barriers for women, reinforcing power structures and organizational cultures that cater to male expectations (36). These challenges highlight systemic issues that demand institutional reforms to foster gender equity, as emphasized by Rathgeber (23).

The individuals who participated in the current study expressed a growing awareness of the need for change, and are advocating for a shift in the current paradigm both at the individual and collective levels, among women in academia. To facilitate this transformation, several opportunities were identified. These include the implementation of institutional policies explicitly promoting gender equality, and the establishment of specialized gender-focused units within research institutions dedicated to implementing these policies.

This has significant implications, particularly in how domestic responsibilities and career interruptions for family-related reasons can disproportionately affect women's research outputs and career advancement compared to their male counterparts. The study suggests that the limited environmental support for research within Nigerian institutions may have a more pronounced impact on women. This is because women often find it challenging to access sponsored opportunities for capacity development due to their obligations to stay home, even when such opportunities arise (37). For instance, women may be less able to take up research grant opportunities, even when these grants are designed to favor the selection of female researchers, due to the caregiving responsibilities they bear. These interruptions to capacity building and empowerment opportunities during the early stages of a woman's career can be difficult to overcome in later years, leading to a widening gender competency gap. The failure to recognize years spent managing households as valuable managerial skills and the insufficient acknowledgment of home caregiving as valuable work skills contribute to women lagging in assessments of their suitability for leadership roles.

In essence, the study underscores the urgent need for institutional and societal changes to address these deeply entrenched gender disparities within research institutions. The USAID Gender Equality Framework (9) shed light on the pervasive gender disparities within research institutions, that are primarily rooted in deeply ingrained patriarchal values present in both societal and institutional contexts. In addition, study participants' growing awareness of the need for change; the need to establish gender-focused units and promote gender equality policies underscores the importance of policy implementation and structural changes within research institutions. In addition, the revelation that domestic responsibilities and career interruptions disproportionately affect women's research outputs and career advancement reinforces the principles in the USAID Gender Equality Framework (9).

### Navigating gender inequity within research institutions and the role of a supportive environment for female medical and dental researchers

While institutional policies and advocacy can contribute to closing gender gaps in research, complete elimination of these disparities remains unlikely. Implementing effective gender equality policies through gender mainstreaming, is a challenging but valuable endeavor (38). In the context of medical and dental research institutions, gender-sensitive policies enacted by dedicated gender focal units could drive progress toward gender equality in research outputs and the representation of women in senior positions. These policies should promote a gender-sensitive review of criteria for appointments and promotions, recognizing the value of home-management skills as administrative abilities. Such efforts could help bridge the gender gap in managerial positions and encourage men to take on caregiving roles without adverse consequences.

The current study findings align with the focus that organizational cultures can either facilitate or hinder women's career advancement (39). The perpetuation of patriarchal values within medical and dental research institutions will require proactive individual and collective advocacy and action by women for organizations to recognize and accommodate women's work-life balance needs (40), including the competency gap resulting from young researchers' career interruptions, and the need for female researchers to access mentorship programs, capacity-building initiatives within organizations that can enable women navigate their careers (41). In addition, the failure to recognize women's skills in managing households and caregiving as valuable managerial and work skills, respectively, reflects a broader societal issue that institutionalizes harmful discriminatory gender norms, beliefs, and stereotypes that hinders women's advancement, and that needs to be addressed.

Medical Women's Associations are known for their success in advocating for policies benefiting women in clinical practice (42). They may need to also focus on addressing gender equality challenges in research. Establishing advocacy groups within academia, involving both women and supportive male allies, could be instrumental in championing gender equality and reshaping traditional notions of masculinity. Advocacy involves a combination of individual and collective actions aimed at gaining political commitment, societal acceptance, and systemic support for specific goals (43). While effective, advocacy can be complex, especially for those with limited resources and influence. Further research is needed to assess how gender equality within research institutions contributes to broader sustainability objectives.

Advocacy, while impactful, often brings about change gradually. In contrast, mentorship, especially when led by women mentoring other women and with the support of male allies, has the potential to drive more rapid transformation in the research context (44). Female mentors play a pivotal role in inspiring other women, fostering a sense of belonging and confidence. Institutionalizing mentorship within research organizations or professional associations helps with taking proactive steps to empower women to engage actively in research alongside their peers. However, mentorship strategies need to be gender-sensitive, and not inadvertently reinforce existing gender inequalities or hierarchies (45). Providing mentors with training in gender-sensitive mentorship practices can mitigate these risks.

The feminist institutionalization analysis highlights the need to embed gender considerations in institutional structures and practices making it less vulnerable to shifts in leadership or external pressures. It recognizes that achieving gender equality often requires persistent efforts to challenge and transform entrenched gender norms, including challenging traditional notions of masculinity within research contexts. Advocacy to change traditional notions can, however, be complex, particularly for those with limited resources and influence. The study findings on the need for gender-sensitive policies, dedicated units, advocacy, mentorship, and the acknowledgment of the complexities involved in achieving gender equality also aligns well with the principles and approaches outlined in the USAID Gender Equality Framework (9). Institutional change is often met with resistance and requires sustained efforts. Supportive networks and alliances are also needed to drive gender equality initiatives. Further research is needed to understand the broader impact of gender equality within research institutions on sustainability goals in view of the need to develop a comprehensive plan to address the gender gaps identified.

### Conceptual framework to study gender inequity in scientific research in West Africa

**Figure 1:**
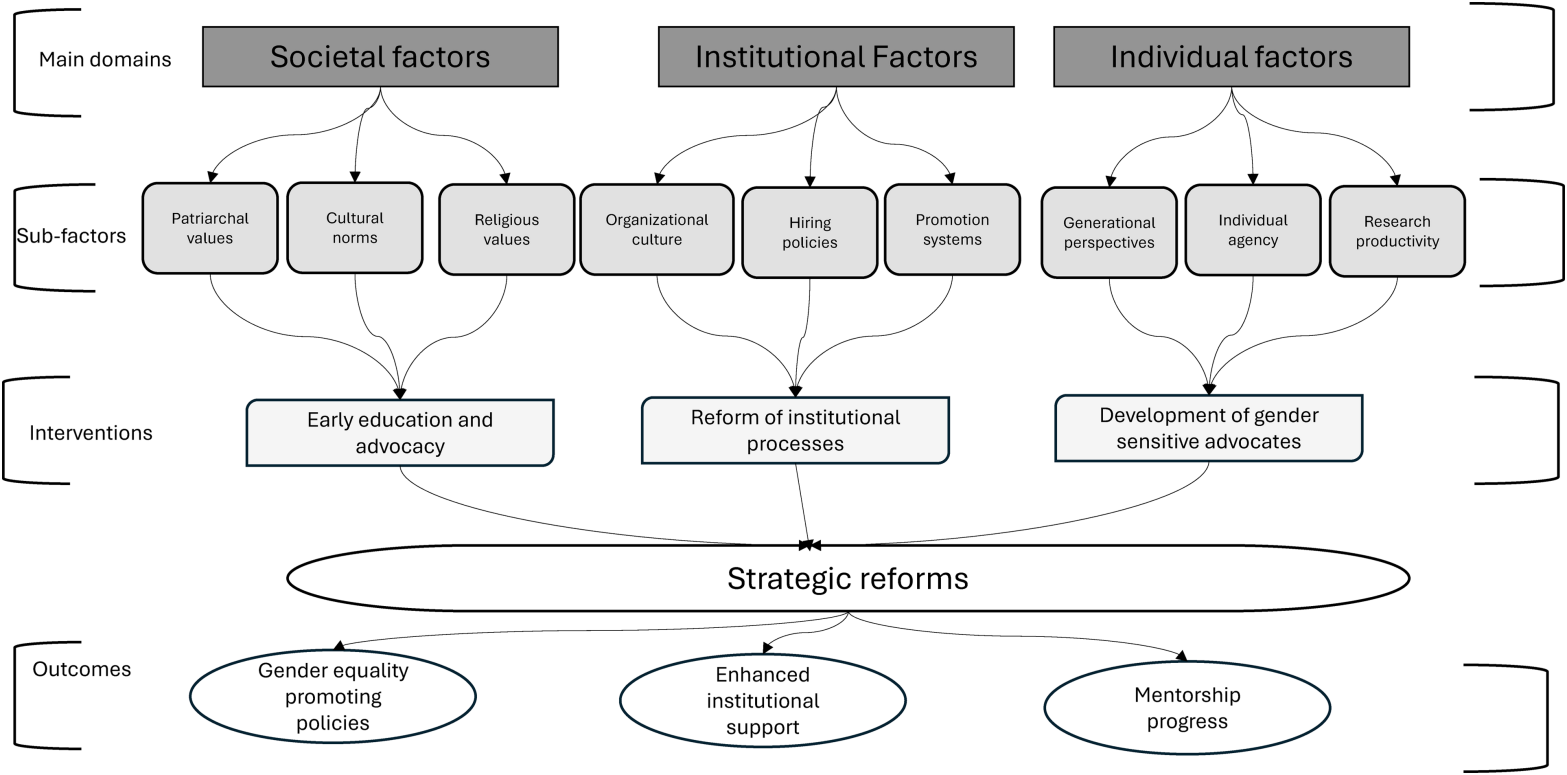
Conceptual framework for the study of gender inequality among medical and dental academia in West Africa.

Figure 1 depicts the conceptual framework for this study. It outlines three core research and action domains—societal, institutional, and individual factors—and their sub-factors, alongside targeted interventions influencing gender inequality, proposed targeted recommendations and expected outcomes. First, the patriarchal societal, cultural, and religious values shape gender roles, restricting women's career advancement. Addressing these barriers requires early education and advocacy to promote gender-sensitive attitudes. Second, institutional policies and organizational culture can further limit female leadership and research productivity, making it essential to reform hiring, promotion, and mentorship processes for inclusivity. Third, individual and generational perspectives influence advocacy, with younger individuals recognizing inequality more readily. Fourth, research productivity and leadership disparities can be addressed through mentorship and training programs for early-career researchers that foster the development of gender-sensitive advocates.

The framework emphasizes the interconnectedness of societal norms, institutional dynamics, and individual agency in shaping gender disparities. It aligns with the gender-transformative program framework, which demonstrates how changes in one part of a societal system can ripple outward, influencing networks and diffusing gender-related outcomes beyond immediate beneficiaries. Norm-shifting programs play a crucial role in reinforcing structural changes, ensuring lasting transformations, and expanding gender-transformative initiatives to achieve widespread societal norm change (46). This underscores the necessity of multi-level actions to address gender inequality in medical and dental academia in West Africa, recognizing that interventions targeting any of the subfactors in Figure 1 can spark meaningful change. Sustained efforts can drive reforms, ultimately achieving the outcomes outlined in the conceptual framework.

Gender inequality and health are deeply interconnected, underscoring the need for programs that transform gender dynamics and norms (47). Such programs must address institutional systems and social structures that distribute power and privilege while simultaneously deconstructing gender norms at the individual level (48). Empowering young leaders and cultivating institutional gender experts to drive inclusivity and advocate for meaningful change in the medical and dental academia in West Africa could be a strategic approach based on the findings of the current study.

Youth-led initiatives are recognized as powerful drivers of gender equality, women's rights, and empowerment, fueled by their passion, creativity, and determination to challenge societal norms and shape a more inclusive future for women and girls (49). However, the transformative potential of young male and female academics in medical and dental fields remains underappreciated. There is a pressing need to inspire and mobilize young academics to actively address gender inequality in their institutions. This would require creating platforms for collaboration, providing mentorship and leadership training, and fostering an enabling environment that promotes gender-reform actions (50). In addition, equipping young academics with the tools and resources to identify and address structural and cultural barriers to equity would empower them to drive meaningful and sustainable change within their institutions. Addressing restrictive gender norms and practices that could hinder the effectiveness of interventions is an essential step in addition to individual level interventions intended to achieve empowerment outcome (51).

The anticipated outcomes include strengthened institutional support, actionable gender-equality policies, and mentorship programs to empower future female leaders in medical and dental academia across West Africa. These efforts are expected to lead to secondary outcomes such as greater representation of women in leadership roles, improved retention and career advancement for female academics, reduced gender disparities in research productivity, and the creation of a more inclusive and equitable academic environment that benefits all members (52, 53). However, achieving meaningful change may be slow without building alliances with broader social justice movements. Accelerating progress on gender inequality in medical and dental academia will also require active collaboration with men and boys to challenge patriarchal norms through intersectional, antipatriarchal social action (54). This transformative social action must be informed by robust evidence and tailored to the West African context, as indicated by findings from research underpinning this theoretical analysis.

The evidence on gender inequality within medical and dental academia in West Africa remains limited, revealing a significant gap in the global gender equality response. Investing in context-specific gender equality initiatives is more likely to be effective, as these actions address the unique cultural, institutional, and societal factors that shape gender dynamics in the region. Context-specific interventions can better identify and address the root causes of inequality, ensure they are relevant to local needs, and encourage greater stakeholder support, leading to more sustainable and impactful outcomes. While addressing gender inequality does not have to begin with targeting its root causes, it should ultimately aim to eliminate them (55). Moreover, gender inequality in medical and dental academia often manifests in more subtle and implicit forms, necessitating targeted gender policies that address these underlying causes (55). These further highlights the need for institutional reforms beyond individual empowerment efforts to tackle gender inequality in this context.

### Study limitations

Although the study provides valuable insights into gender inequities in medical and dental academia in West Africa, it is limited by several factors. The contextual application of theoretical frameworks, developed in broader settings, may not fully capture localized socio-cultural dynamics. Over-reliance on self-reported data introduces potential biases, while limited regional coverage of the study also restricts the generalizability of findings. The study also lacks an intersectional analysis of factors like race and class.

In addition, the study offers limited exploration of men's roles as advocates for gender equity and insufficient analysis of empowerment dynamics beyond academia. Also, theoretical frameworks were used statically, without examining their adaptability to evolving gender dynamics. Addressing these limitations through broader regional and disciplinary coverage, intersectional analysis, and engagement with male allies can enhance future research. This would provide a more comprehensive understanding and actionable strategies for fostering gender equity in West African academia.

## Conclusions

This study highlights the complex interplay of societal, cultural, and institutional factors contributing to gender inequality in scientific research within West African medical and dental research institutions. Rooted in patriarchal norms and institutional biases, these disparities manifest as underrepresentation of women in leadership roles, limited research productivity, and constrained agency in career advancement. Despite these challenges, women researchers are demonstrating resilience and advocacy, though systemic support remains insufficient.

The findings emphasize the urgent need for culturally sensitive strategies, including reforms in institutional policies, mentorship programs, and advocacy initiatives to address visible and hidden barriers. Promoting early education to challenge gender norms, empowering younger leaders, and fostering gender-sensitive organizational cultures are critical for meaningful change. Additionally, enhancing access to resources and recognizing domestic management as a valuable skill for leadership can support the progression of women in academia.

By leveraging these insights and integrating evidence-based approaches, research institutions in West Africa can take strategic steps toward creating equitable and inclusive academic environments, ultimately bridging gender gaps and enhancing research outcomes.

## Data Availability

The original contributions presented in the study are included in the article/Supplementary Material, further inquiries can be directed to the corresponding author.

## Footnotes

^1^Merluzzi J, Phillips DJ. Leadership and the single woman penalty: a role expectations account of promotion penalties toward female professionals. Available online at: https://sites.insead.edu/facultyresearch/research/file.cfm?fid=58706 (accessed December 27, 2024).
